# Correspondence between Dr. Jenner and Dr. Marcet

**Published:** 1803-05-01

**Authors:** Alexander Marcet

**Affiliations:** London


### *To the Editors of the Medical and Physical Journal.*

Gentlemen,

I TAKE the liberty of sending you enclosed an extract of a correspondence between Dr. Jenner and myself, containing some observations on the subject of Vaccine Inoculation, which Dr. Jenner has given me leave to communicate to the Public through your valuable Journal. I feel a particular pleasure in collecting facts and observations from a man who unites to the brilliant claims of discovery, the merit (perhaps still greater) of candid enquiry and indefatigable observation; and I should think myself truly blamable if I did not make the Public participate in remarks coming from such high authority upon so interesting a subject.

*London,* I am, &c.

*April* 20, 1803. ALEXANDER MARCET.

---

### *Extract of a Letter from Dr.* Jenner *to Dr.* Marcet, *dated London, Feb.* 23, 1803.

"THE Copenhagen report on the subject of Vaccination is equally satisfactory with that of other countries. Nevertheless theless it strikes me, that the virus they employ there has probably undergone some change; a change not sufficient to deprive it of those specific qualities which render it capable of destroying in the constitution the susceptibility of the contagion of Small-pox, but yet sufficient to effect some little alteration or derangement in its properties; for it is said in the report, "Those inoculated frequently have eruptions." Now, in this country, out of the vast number I have inoculated (a number in conjunction with my nephews but little short of ten thousand), I neither see nor hear of cases of eruption, except those trifling appearances I have noticed in one of my publications, a kind of rash somewhat resembling that from teething; but even this is very rare. Similar occurrences to those in Denmark, have taken place both in this country and in many others. In Hanover the same thing took place; and in a district in Scotland, I have in two or three instances seen perfect vaccine pustles, one or two upon a subject.

"The origin of this deviation in my opinion is this; the use of the Vaccine fluid, when taken so late from a pustule that it was *begining* to undergo a change, which rendered it incapable of exciting the same effect *precisely* as if it had been taken at an earlier period. The truth of this theory seems to have been exemplified in many instances. There may be every gradation in the degeneracy of the virus from that point where it is capable of simply producing no further change in the pustule subsequently propagated, down to that which produces the compleatly spurious pustule; for it must be remarked, that a pustule in a state of degeneracy will produce another of the same kind. This indeed was exemplified in the case of the Vienna nobleman, on whose arm a spurious pustule was produced, from which a succession of the same kind sprung up at Geneva.

"The same circumstances took place in America in the early days of Vaccination there, before its phænomena were well understood by Professor Waterhouse; and it has happened, I am sorry to say, many times in this country. That one degeneracy will create another we have an instance not uncommon in the purulent pustule. I frequently find when a child has a scabby face, especially if it be accompanied with tinea capitis, or papillous eruptions, that the purest vaccine virus will produce a pustule that will never contain limpid matter, but throughout all its stages its contents will be purulent. If I inoculate from this source, almost to a certaintity I produce a similar pustule:

 the the scab which succeeds is not hard and dark coloured like the common Vaccine scab, but soft and of an amber colour, like that of the Small-pox, and the whole progress of the disease is accelerated."

---

*Extract of a Letter from Dr.* MARCET *to Dr. Jenner, dated London, March* 25, 1803,

" I BEG leave to send you enclosed two little plates of glass, intended for the preservation of Vaccine virus, which appear to me somewhat better adapted to the purpose than any of the means that have hitherto come to my knowledge.

" Glass, in every point of view, seems to be the substance best calculated for the purpose, as it is of all ingredients the least likely to be acted upon by the virus, or to produce by its own agency any chemical alteration whatever. The little bottles in which matter is preserved on an elongated glass stopper, as well as some other ingenious contrivances for the preservation of virus, answer this purpose but imperfectly ; and even the small plates of glass which you have brought into use, and which offer by far the simplest as well as the most effectual method that has hitherto been used, cannot be said to exclude the air entirely, since it is obvious, that whenever a foreign body is placed between two polished surfaces, it is impossible that these two surfaces should come into perfect contact.

" The hope of removing such objections, and of reconciling to the use of glass some medical men, who seem to have taken a sort of dislike to it, and whose authority may have a considerable share in framing the general plan of instruction which is at this moment in contemplation, induced me to try whether the means hitherto employed were not susceptible of some further improvement.

" The enclosed plates of about three-quarters of an inch square, are made of pretty thick glass, perfectly flat, and ground rough on one side. In the centre of the rough surface, you will observe a round spot about three-eighths of an inch in diameter, which has been polished afterwards. It results from this disposition, that, when you apply the two rough surfaces to each other, the plates come every where into perfect contact, with the exception of an extremely small space left between the two polished spots; and if a drop of matter be spread between these spots, it is there as effectually preserved from any access of of air as if the glass was hermetically sealed; and as this drop of virus just fills the space allowed, no sensible quantity of air can either be lodged or penetrate into that space.

"The manner in which the virus is deposited on the glass is nearly this: After a puncture or two have been made in the pustule, and a quantity of fluid allowed to ooze out of it, till it appears in the form of a distinct drop, the plate of glass is gently applied to the pustule, so as to bring the virus in contact with the polished spot above described. The pustule may then be punctured afresh once or twice, and the plate again applied to it, until a drop has been collected on its surface sufficient to fill exactly the small recess allowed for it; and on applying the two plates against each other, the drop of virus will be seen to spread a little, till its edges all round reach the rough surfaces by which it is confined. The strong adhesion of the two plates of glass will prove how perfectly the air is excluded.

"It has been suggested, (although, I believe, without any positive foundation) that *light* may act upon virus through glass; but this objection might with the greatest facility be removed, merely by spreading a little opaque black paint over the outside of the plates. It will be found that ground glass has also the advantage of making the plates in some degree stick together, whilst those made of plain glass are exceedingly apt to slide over each other; a piece of paper will therefore be perfectly sufficient to confine these plates; but if an apparatus still more convenient was required, it might probably be procured, on the same principle, by having a glass socket, ground to a glass stopper filling exactly the whole of its capacity, with the exception only of a small polished spot on the stopper itself, where the matter could be deposited. This, however, would be more or less expensive, whilst the other apparatus proposed (which was cut and ground for me at Mr. Parker's glass-shop) cost only one shilling.

"Glass, particularly if the plates be made of tolerable thickness, has the additional advantage of being a very bad conductor of heat, and of thus defending matter more effectually against changes of temperature, the importance of which you have strongly pointed out. It is obvious, for instance, that if a person stands very near the fire with matter in his pocket, both on a lancet and on glass, the lancet will become burning hot, before the internal surface face of the glass has suffered scarcely any increase of temperature.

"It has been objected, that glass is apt to blunt the lancet; but if this should be found a material inconvenience, it might easily be removed by previously diluting and scraping the matter with another lancet, kept for that purpose; or, what would be still better, with some ivory instrument.

"I must at the same time confess, that in submitting to you these remarks, I reason rather from theory, and from the observations of others, than from any extensive personal experience; but as far as my own observation goes I may say, that I have found matter kept on glass uniformly successful; whilst I have repeatedly failed with virus kept on threads or lancets; and I have also sent to Copenhagen, matter simply enclosed in a letter between two plates of glass, which has succeeded perfectly.

"I am afraid, dear Sir, that the length of this letter requires some apology. I am perfectly sensible that the few observations it contains, can only deserve your attention from the importance of their object, and it is merely upon that ground that I have presumed to submit them to your consideration."

*Extract of* Dr. JENNER's *Answer to* Dr. MARCET, *dated April* 6, 1803.

"——— I am obliged to you for the glasses you sent me for the preservation of Vaccine matter. They are better constructed for the purpose than any I have yet seen. What substance can be so good as glass for the conveyance of a virus so peculiarly delicate and liable to decomposition, to distant parts? I have known the Vaccine virus in full possession of all its specific properties, when preserved in this way, seventeen weeks; but it must be observed, that whatever substance be made use of, the quantity of fluid collected should not be too small. At the expiration of three months, some Vaccine virus which I had accumulated on a quill, and suspended in a half ounce phial, was used with complete success. By the way, from this virus the first patient ever inoculated in the metropolis received the Vaccine disease. It was used at my request by Mr. Cline."

